# Health-Related Quality of Life, Psychological Health, and Patient-Reported Outcomes of Amyotrophic Lateral Sclerosis Patients in China

**DOI:** 10.3390/brainsci15070696

**Published:** 2025-06-28

**Authors:** Dilip Dhakal, Congzhou Chen, Bo Zhang, Guanqiao Li

**Affiliations:** 1 Vanke School of Public Health, Tsinghua University, Beijing 100084, China; drdilipdhakal@gmail.com (D.D.); congzhou.chen20@gmail.com (C.C.); 2 Guangzhou Magpie Pharmaceuticals Co., Ltd., Guangzhou 510700, China; 3 Institute for Healthy China, Tsinghua University, Beijing 100084, China; 4 Department of Pharmacy, Chinese Academy of Medical Sciences & Peking Union Medical College, Beijing 100730, China

**Keywords:** amyotrophic lateral sclerosis (ALS), health-related quality of life (HRQoL), psychological health, patient-reported outcomes (PROs)

## Abstract

**Objectives:** This study explored the health-related quality of life (HRQoL), psychological health, and patient-reported outcomes (PROs) of patients with amyotrophic lateral sclerosis (ALS) in China, providing insights for enhancing clinical care. **Methods:** A cross-sectional study was conducted among Chinese ALS patients between February and May 2024. Demographics, clinical characteristics, and PROs were assessed. HRQoL and psychological health were evaluated via the 5-item amyotrophic lateral sclerosis assessment questionnaire (ALSAQ-5) and the 4-item patient health questionnaire (PHQ-4), respectively. Spearman’s rank correlation, multiple linear regression, and the Kruskal–Wallis H test were used to analyze associations between clinical factors, HRQoL, and psychological health. **Results:** A total of 237 participants aged 46–65 years (63.3%) were included. The mean ALSAQ-5 score was 64.86±19.34, indicating an impaired HRQoL, whereas the mean PHQ-4 score (5.82 ± 4.10,) suggested varied degree of anxiety and depression. Age, disease duration, ALS severity, fatigue, stress, and pain severity, and respiratory support were significantly associated with HRQoL (*p* < 0.05). Age, stress severity, and pain severity were significant predictors of psychological distress (*p* < 0.01). Patients reported diagnostic delay, profound lifestyle changes (96.4%), fear of paralysis (84.8%), and death (49.8%). Most patients (80.6%) expressed a strong desire to stop ALS progression, prioritizing treatments that improve breathing, muscle weakness, swallowing, and mobility issues. **Conclusions:** ALS profoundly impacts patients’ HRQoL and psychological health. Integrating PROs into clinical care strategies is crucial for improving patient outcomes and guiding treatment priorities.

## 1. Introduction

Amyotrophic lateral sclerosis (ALS) is a rare, devastating neurodegenerative disorder, characterized by the selective death of both upper and lower motor neurons [1,2]. This leads to progressive muscle weakness, atrophy, and difficulties in swallowing and breathing, ultimately resulting in death due to respiratory failure [3,4,5,6,7], with a median survival of 2–5 years following the onset of initial symptoms [8]. Currently, there is no cure for ALS, and approved medications provide only modest benefits without halting disease progression [9]. Therefore, the primary focus of ALS management has focused on improving health-related quality of life (HRQoL) and psychological health through multidisciplinary supportive care [9,10].

Research on the HRQoL and psychological health of ALS patients in China remains relatively limited. Currently, HRQoL assessments for ALS patients are mostly carried out in clinical research settings using broader scales such as the 40-item amyotrophic lateral sclerosis assessment questionnaire (ALSAQ-40) [11]. Shorter, more efficient scales, such as the 5-item amyotrophic lateral sclerosis assessment questionnaire (ALSAQ-5) [12] and the 4-item patient health questionnaire (PHQ-4) [13], could be valuable for large-scale HRQoL assessments and psychological health screening. However, they are not yet widely adopted in clinical practice. Consequently, the comprehensive assessments of HRQoL and regular psychological monitoring of ALS patients remain limited.

The use of patient-reported outcomes (PROs) in rare disease research and clinical practice holds significant potential for enhancing patient care and improving clinical outcomes [14]. Given the substantial impact of ALS on daily life, there has been increasing research interest in understanding PROs, which provides direct insights into patients’ perceptions of their health status, disease burden, and treatment priorities [4,15]. Despite growing interest worldwide, data on PROs among ALS patients in China remain limited. To our knowledge, this is the first study to report the PROs of ALS patients in China.

This study aim to evaluate HRQoL, psychological health, and PROs in Chinese ALS patients.

## 2. Materials and Methods

### 2.1. Study Design and Participants

This study employed a cross-sectional, survey-based research design and was conducted in China between February 2024 and May 2024. The inclusion criteria were as follows: adult Chinese individuals aged ≥ 18 years, diagnosed with ALS, and able to answer survey questions on their own or with the assistance of a caregiver. All participants provided informed consent prior to their enrollment in the study. This study was approved by the Tsinghua University Science and Technology Ethics Committee (THU01-20240014).

### 2.2. Demographics and Clinical Variables

Demographic and ALS-related data, including age, sex, ALS diagnosis and severity, time since first symptom, ALS onset-to-diagnosis time, and treatment, were collected.

### 2.3. ALS Severity Assessment

ALS severity was assessed using the Japanese ALS Severity Classification scale, which categorizes patients into five grades based on their level of functional impairment [16]. The scale ranges from grade 1, representing the least impairment, to grade 5, indicating the most severe disability, with each grade defined by specific criteria related to the patient’s ability to work, live independently, perform daily activities, and respiratory insufficiency and the need for medical interventions.

### 2.4. Fatigue, Stress, and Pain Assessments

Fatigue levels were evaluated to quantify the self-assessed average fatigue level experienced over the previous two weeks. Fatigue severity was categorized into four levels: not fatigued, mildly fatigued (fatigue relieved by physical rest), moderately fatigued (limiting instrumental ADLs), and very fatigued (limiting self-care ADLs) [17].

Stress levels were evaluated to quantify the self-assessed average stress level experienced over the previous two weeks. Stress severity was categorized into five levels: none, mild, moderate, high, and maximum [17].

The numeric pain rating scale (NPRS) was used to evaluate the pain intensity experienced over the previous two weeks of pain [18,19]. The NPRS score ranging from 0 to 10 allows patients to score their level of pain on a scale from 0, which indicates “no pain at all,” to 10, which is “the worst possible pain.” Pain severity is graded as an NPRS score of 0 (no pain); 1–3 (mild pain, discomfort, and annoying but does not interfere with most ADLs); 4–6 (moderate pain, interferes with ADLs and may require medication or other interventions); and 7–10 (severe pain, significant interference with ADLs and requiring prompt and aggressive pain management strategies) [18,20].

### 2.5. HRQoL and Psychological Health Assessments

The self-administered ALSAQ-5 scale was used to assess the HRQoL of ALS patients by focusing on disease-specific symptoms [21]. This streamlined version maintains statistical parity with the ALSAQ-40 in gauging HRQoL, but boasts a higher response rate, rendering it exceptionally suitable for real-world applications. The ALSAQ-5 comprises five domains: physical mobility, ADLs and independence, eating and drinking, communication, and emotional functioning. Each domain comprises one question with five possible answers. Each domain is rated on scale of 0–4 (never—0, rarely—1, sometimes—2, often—3, always or unable do at all—4). The individual percentage score for each question is calculated using the formula: x1 = (*n*/5) × 100, where *n* is the score provided [21]. The total ALSAQ-5 score was calculated as the mean of the percentage scores of all five domains. The cumulative score, ranging from 0 to 100, reflects an individual’s health state, with 100 representing the worst quality of life; specifically, scores of 0–19 indicate “never,” 20–39 “rarely,” 40–59 “sometimes,” 60–79 “often,” and 80–100 indicate that problems are “always present” or the activity is “unable to be do at all” [21,22].

The self-assessed PHQ-4 scale was used for screening psychological health (depression and anxiety) in patients with ALS over the last two weeks [13]. The PHQ-4, a concise version of the PHQ-9, integrates the PHQ-2 and GAD-2 scales, making it particularly useful for providing rapid and efficient psychological health screening for ALS patients in clinical settings. The PHQ-4 scale comprises two questions each for anxiety (feeling nervous, anxious, or on edge; not being able to stop or control worrying) and depression (feeling down, depressed or hopeless; little interest or pleasure in doing things). Each question is rated on a points scale of 0–3 based on symptoms frequency as 0 = not at all, 1 = several days, 2 = more than half the days, and 3 = nearly every day with a total maximum score of 12 points. Scores ≥ 3 on the anxiety subscale (items 1–2) are indicative of possible anxiety disorders, whereas scores ≥ 3 on the depression subscale (items 3–4) suggest possible depressive disorders [13].

### 2.6. Patient-Reported Outcomes Assessments

The PROs were assessed using a structured questionnaire. PROs included (1) self-reported symptoms; (2) patient priorities for desired improvements in ALS symptoms or functional impairment; (3) use of approved therapies; (4) the use of symptomatic treatments; (5) patient perception of how well their current treatment plan manages ALS; (6) self-reported lifestyle changes; (7) significant downsides associated with ALS diagnosis and current treatments as reported by patients; and (8) future fears expressed by the patients.

### 2.7. Statistical Analysis

The reliability of the ALSAQ-5 and PHQ-4 was assessed Cronbach’s alpha, and internal validity was assessed using Kaiser–Meyer–Olkin and Bartlett’s test [23,24].

Categorical data are presented as frequencies and percentages, whereas continuous data are expressed as mean ± SD for normally distributed variables or medians with interquartile ranges for distributed variables.

Associations between demographic and clinical variables with ALSAQ-5 and PHQ-4 scores were examined using Spearman’s rank correlation coefficient (r_s_). For subgroup analyses, the Kruskal–Wallis H test was used for non-parametric comparisons of continuous variables across multiple groups.

Multiple linear regression analysis was performed to identify the independent predictors of ALSAQ-5 and PHQ-4 scores. Variables with significant correlations (*p* < 0.05) in the univariate analysis or those deemed clinically relevant were included in the multivariate model. A stepwise forward method was used for variable selection. All the statistical analyses were performed in R (version 3.5.6).

## 3. Results

### 3.1. Demographic and Clinical Variables

A total of 237 patients with ALS were included, with the majority aged 46–65 years (63.3%) and male (58.6%). Sporadic ALS was diagnosed in 59.5% of patients, and 37.6% were uncertain of their ALS type. Most patients (79.3%) had experienced symptom onset within the last three years. Over one-third of the ALS patients (34.9%) were diagnosed more than one year after symptom onset, indicating a notable delay in diagnosis for a significant portion of the cohort.

Moderate-to-severe fatigue, stress, and pain were reported by 52.3%, 49.8%, and 31.6% of patients, respectively. The demographic and clinical characteristics of the participants are shown in Table 1.

### 3.2. HRQoL and Psychological Health Assessment

The mean HRQoL score, as measured by the ALSAQ-5, was 64.86 ± 19.34, indicating an overall impaired quality of life in these patients. The mean score for psychological health, as measured by the PHQ-4, was 5.82 ± 4.10, suggesting that these patients may have varying degrees of anxiety and/or depression (Table 1).

In the reliability and internal consistency assessments, the ALSAQ-5 showed an acceptable Cronbach’s alpha coefficient of 0.761. The PHQ-4 scale showed excellent internal consistency, with a Cronbach’s alpha coefficient of 0.934. Both scales were internally validated with a significant Bartlett test (*p* < 0.001) and a high Kaiser–Meyer–Olkin measure of 0.860, suggesting that an adequate sample size and the data collected were suitable for factor analysis.

### 3.3. Clinical Factors Associated with HRQoL and Psychological Health

To better understand the factors influencing HRQoL in ALS patients, significant correlations were observed between the ALSAQ-5 score and age (r_s_ = 0.169, *p* = 0.009), time since first symptom onset (r_s_ = 0.274), ALS severity (r_s_ = 0.728), fatigue severity (r_s_ = 0.473), stress severity (r_s_ = 0.480), pain severity (r_s_ = 0.425), and respiratory support (r_s_ = 0.464) (all *p* < 0.001).

The PHQ-4 score was associated with age (r_s_ = 0.181, *p* = 0.005), ALS severity (r_s_ = 0.399), fatigue severity (r_s_ = 0.416), stress severity (r_s_ = 0.593), pain severity (r_s_ = 0.393) (all *p* < 0.001), and respiratory support (r_s_ = 0.197, *p* = 0.002). The time since first symptom onset and the time from symptom onset to diagnosis were not significantly correlated with the PHQ-4 scores (Table 2).

### 3.4. Subgroup Analyses

ALSAQ-5 scores varied significantly across subgroups for time since first symptom onset, ALS severity, respiratory support, pain, fatigue, and stress severity (all *p* < 0.001). However, no significant differences in ALSAQ-5 scores were found for age, sex, type of ALS diagnosis, time from symptom onset to diagnosis, or ALS treatment drugs.

Age (*p* = 0.023), respiratory support (*p* = 0.004), ALS severity, pain, fatigue, and stress severity (all *p* < 0.001) were associated with significant differences in the PHQ-4 scores. No significant differences in the PHQ-4 scores were observed for sex, type of ALS diagnosis, time from first symptom onset, time from symptom onset to diagnosis, or ALS treatment drugs. All subgroup analyses are shown in Table A1.

### 3.5. Predictors of HRQoL and Psychological Health in ALS Patients

Multiple linear stepwise regression analyses were conducted to identify the factors associated with the ALSAQ-5 and PHQ-4 scores (Table 3). Age (β = 0.1785, *p* = 0.0405), time since first symptom onset (β = 0.2241, *p* = 0.0003), fatigue severity (β = 4.03, *p* = 0.0065), stress severity (β = 4.8396, *p* = 0.0001), and pain severity (β = 1.7834, *p* < 0.0001) were positive predictors of low HRQoL in ALS patients (adjusted R^2^ = 0.3832, *p* < 0.0001).

Age (β = 0.0464, *p* = 0.0105), stress severity (β = 2.0053, *p* < 0.0001), and pain severity (β = 0.2627, *p* = 0.0045) were identified as positive predictors of greater psychological distress in ALS patients (adjusted R^2^ = 0.3824, *p* < 0.0001).

### 3.6. Patient-Reported Outcomes

#### 3.6.1. Common and Desired Improvement in ALS Symptoms

**Figure 1:**
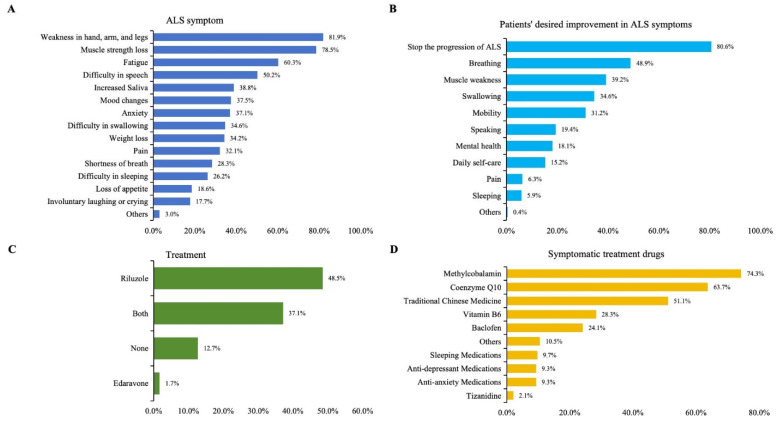
(**A**) Self-reported symptoms in ALS patients. (**B**) Patient priorities for desired improvements in ALS symptoms or functional impairment. (**C**) Use of approved therapies among the surveyed ALS patients. (**D**) The use of symptomatic treatments reported by the patients.

Common ALS symptoms include muscle atrophy and weakness, difficulty in speech and swallowing, fatigue, and weight loss. The most frequently reported ALS symptoms were weakness in the hands, arms, and legs (81.9%), loss of muscle strength (78.5%), fatigue (60.3%), difficulty in speech (50.2%), increased saliva (38.8%), weight loss (34.2%), mood changes (37.6%), and anxiety (37.1%) (Figure 1A).

When asked about the desired improvements in ALS symptoms or functional impairments, the majority of the patients (80.6%) expressed a strong desire to stop ALS progression. Specifically, patients prioritized treatments that could improve their breathing (48.9%), muscle weakness (39.2%), swallowing (34.6%), and mobility (31.2%) (Figure 1B).

#### 3.6.2. ALS Medications and Symptomatic Treatments

The medications used for treating ALS by the greatest proportion of patients was riluzole (48.5%) alone, both riluzole and edaravone (37.1%), and edaravone alone (1.7%). No ALS-specific drugs were used by 12.7% of the patients (Figure 1C). Symptomatic treatments included methylcobalamin (74.3%), coenzyme Q10 (63.7%), traditional Chinese medicine (51.1%), vitamin B6 (28.3%), and baclofen (24.1%) (Figure 1D).

#### 3.6.3. Treatment Effectiveness and Disease Challenges

**Figure 2:**
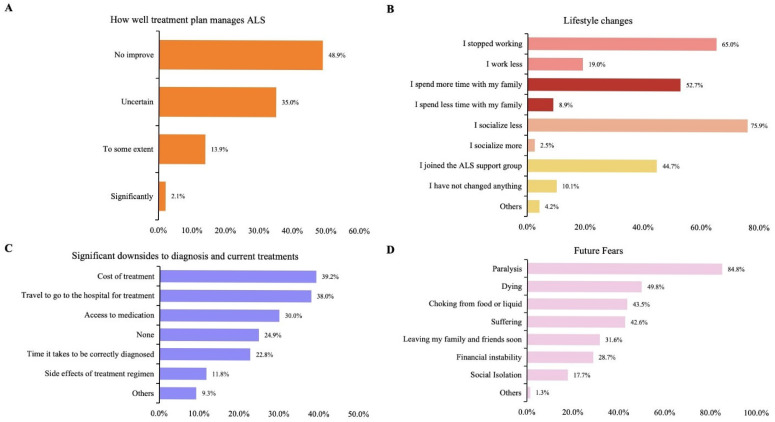
(**A**) Patient perception of how well their current treatment plan manages ALS. (**B**) Self-reported lifestyle changes experienced by patients with ALS following their diagnosis. (**C**) Significant downsides associated with ALS diagnosis and current treatments as reported by patients. (**D**) Future fears expressed by the patients.

Regarding the response to the question “How Well Treatment Plan Manages ALS,” nearly half of the patients (48.9%) reported no change in their symptoms or disease progression, whereas 35% were uncertain about the effectiveness of their current treatment. Only 2.1% believed that their current treatment significantly managed their disease, indicating the urgency of developing more targeted therapies for ALS (Figure 2A).

The most profound impacts on lifestyle included reduced socialization (75.9%), cessation of employment (65%), increased time spent with family (52.7%), and joining an ALS support group (44.7%) (Figure 2B).

Patients identified the cost of treatment (39.2%), travel to the hospital for treatment (38.0%), and access to medication (30%) as significant downsides of their current ALS treatment (Figure 2C).

In response to the question, “What is your greatest fear about the future?” Patients most frequently reported paralysis (84.8%), death (49.8%), choking on food or liquids (43.5%), suffering (42.6%), and prematurely leaving family and friends (31.6%) (Figure 2D).

## 4. Discussion

This study highlights the impaired HRQoL and significant impact on the psychological health in ALS patients, as measured by the ALSAQ-5 and PHQ-4 scales. Age, fatigue severity, stress severity, and pain severity were the key predictors of negative health outcomes. The PROs further emphasized the challenges in the diagnosis and management of ALS. More than one-third of patients are uncertain about their ALS subtype, and diagnostic delays are frequently reported, suggesting potential gaps in the early diagnosis and classification of ALS subtypes, which are essential for guiding appropriate treatment decisions. Current treatments only show minimal effectiveness in managing ALS symptoms, and patients express a strong desire for therapies that can slow disease progression by targeting breathing, muscle strength, and mobility. Moreover, our findings support the use of the ALSAQ-5 and PHQ-4 as valid instruments for assessing HRQoL and screening psychological health in ALS patients.

Our study revealed that Chinese patients with ALS experience a moderate degree of HRQoL deterioration, as measured by the ALSAQ-5 scale. Multiple regression analysis identified age, disease duration, fatigue, stress, and pain severity were independent predictors of HRQoL in these patients. Age was identified as a significant predictor of low HRQoL in these patients, which is consistent with previous studies suggesting that older individuals may encounter greater functional limitations [25,26]. Most patients had a disease duration between 12 months and 5 years, and a longer disease duration was associated with a lower HRQoL. However, a large cohort study found no such association, highlighting the variability in ALS progression and its impact on HRQoL [27]. Fatigue, stress, and pain severity were identified as significant, independent factors affecting HRQoL in our study. Fatigue, often presenting as general exhaustion, and pain, which limits daily activities, are both common and strongly linked to low HRQoL [28,29,30]. Stress, exacerbated by physical symptoms, further contributes to emotional burden. These findings highlight the need for targeted management of fatigue, stress, and pain to improve the overall HRQoL in ALS patients.

The PHQ-4 self-report scale was used to assess psychological health, and it was found that these patients might experience anxiety or depression. Physical function loss as the illness worsens causes a daily emotional burden and a greater likelihood of anxiety or depression [31]. Multiple regression analysis revealed that age is a significant predictor of psychological health, with older age associated with increased PHQ-4 scores. However, previous studies have revealed that age is not a risk factor for depression in individuals with ALS, suggesting that age-related comorbidities (rather than age itself) may confound the assessment of psychological distress in older patients [32]. A significant increase in the PHQ-4 score was found as pain and stress severity increased. Our study found that higher pain severity was significantly associated with increased PHQ-4 scores, which is consistent with a study involving 636 ALS patients reported that those experiencing pain had notably higher anxiety levels [33]. Stress is another important factor that contributes to poor psychological health [34]. Fear of dying, suffering, and financial hardship may contribute to increased stress severity. These findings emphasize the importance of early psychological evaluation and support for ALS patients.

The analysis of PROs offers valuable insights into how ALS patients perceive their symptoms, disease burden, and treatment priorities [35,36]. We found that approximately one-third of the patients were diagnosed after 1 year of symptom onset, which differs from the findings of other studies [37,38]. Due to the rarity and heterogeneity of the disease, clinicians often face challenges in making an accurate diagnosis. As the median survival is only 2–5 years, early diagnosis is crucial to maximize the benefits of disease-modifying treatments and improve clinical management [39]. Despite the use of approved medications, such as riluzole and edaravone, only 2.1% of patients believe that their current treatment can manage their disease progression, reflecting dissatisfaction with the available therapeutic options. These patient-reported outcomes align with real-world evidence, as a study in a Chinese ALS cohort also revealed no significant survival benefit from riluzole treatment [40]. These findings further verify the urgent need for more effective and targeted therapies.

The majority of patients expressed a strong desire to slow disease progression, with particular emphasis on improving breathing, muscle strength, swallowing, and mobility, which is consistent with other studies [17,41]. Patients also reported substantial lifestyle impacts, including reduced social interaction and job loss, leading to financial instability and decreased access to healthcare resources, thereby exacerbating the existing burdens faced by patients and their caregivers. ALS patients face emotional challenges such as fear of loss of mobility, choking, fear of death, and separation from loved ones, which is similar to the situation in US ALS patients [17]. These findings emphasize the value of integrating PROs into ALS care to better address patient needs and guide therapeutic development.

### Strengths and Limitations

This is the first study in China to comprehensively evaluate HRQoL, psychological health, and PROs in a relatively large cohort of ALS patients, despite the rarity of the disease. The use of brief scales such as the ALSAQ-5 and PHQ-4 enhances the clinical applicability of our findings. Additionally, it highlights patient priorities and treatment experiences, which contribute to improve patient care and outcomes.

This study has certain limitations. The cross-sectional design of this study restricts the ability to assess changes in HRQoL, psychological health, or PROs over the course of the disease, and it is critical to understand longitudinal changes in ALS because of its progressive nature. Moreover, participant recruitment, primarily through convenience sampling, may have limited the generalizability of our findings. This approach could lead to the underrepresentation of individuals in the most advanced stages of ALS, potentially resulting in an underestimation of the overall disease burden. Future research should prioritize longitudinal studies and use validated tools for the measurement of fatigue and stress severity. This study did not collect data on cognitive impairment. As cognitive impairment may influence the patient’s outcomes in ALS, future research should incorporate cognitive assessments to provide a more comprehensive evaluation.

## 5. Conclusions

This study provides valuable clinical insights for improving ALS care in Chinese patients. The use of brief scales, such as the ALSAQ-5 and PHQ-4, demonstrates their feasibility and relevance for the routine assessment of HRQoL and screening of psychological health in clinical settings. By identifying key predictors of adverse outcomes such as fatigue, pain, and stress, our findings support the need for a multidisciplinary approach to ALS treatment. Clinicians should prioritize early diagnosis to improve patient outcomes. Additionally, the inclusion of PROs underscores the importance of incorporating patient perspectives into clinical decision-making, thereby enabling more patient-centered approaches to ALS care.

## Figures and Tables

**Table 1:** Demographic and clinical characteristics of the ALS patients.

Characteristics	ALS (*N* = 237)	Mean (SD)
	*n* (%)	
Age group		
18–35	15 (6.3)	
36–45	37 (15.6)	
46–55	83 (35.0)	
56–65	67 (28.3)	
≥66	35 (14.8)	
Sex		
Male	139 (58.6)	
Female	98 (41.4)	
ALS type		
Sporadic ALS	141 (59.5)	
Familial ALS	7 (3.0)	
Uncertain	89 (37.6)	
Time since first symptom		
0–12 months	31 (13.1)	
12–18 months	49 (20.6)	
18 months to 2 years	46 (19.3)	
2–3 years	50 (21.1)	
3–5 years	43 (18.2)	
More than 5 years	18(7.6)	
Time from symptom onset to diagnosis		
Within 3 months	11 (4.6)	
3–6 months	54 (22.8)	
6–12 months	90 (38.0)	
12 months–18 months	45 (19.0)	
18 months–2 years	16 (6.8)	
2–3 years	9 (3.8)	
More than 3 years	12 (5.1)	
ALS severity scale		
Grade 1—able to work or perform housework	24 (10.1)	
Grade 2—independent living but unable to work	79 (33.3)	
Grade 3—requiring assistance for eating, excretion, or ambulation	85 (35.9)	
Grade 4—presence of respiratory insufficiency/dysphagia	35 (14.8)	
Grade 5—use of tracheostomy tube, tube feeding or tracheostomy-positive pressure ventilation	14 (5.9)	
Fatigue severity		
Not fatigued	22 (9.3)	
Mildly fatigued	91 (38.4)	
Moderate fatigued	84 (35.4)	
Severe fatigued	40 (16.9)	
Stress severity		
None	29 (12.2)	
Mild	90 (38.0)	
Moderate to high	102 (43.0)	
Maximum	16 (6.8)	
Pain severity		
No pain	67 (28.3)	
Mild	95 (40.1)	
Moderate	55 (23.2)	
Severe	20 (8.4)	
ALSAQ-5 total score		64.86 (19.34)
PHQ-4 total score		5.82 (4.10)

The total ALSAQ-5 score was calculated as the mean of the percentage scores of all five questions in the ALSAQ-5 scale (physical mobility, activities of daily living, eating and drinking, communication, and emotional functioning). The total PHQ-4 score is calculated as the mean of all four questions on the PHQ-4 scale (feeling nervous, anxious, or on edge; not being able to stop or control worrying; feeling down, depressed, or hopeless; and little interest or pleasure in doing things). Pain severity is graded as a numeric pain rating scale score of 0 (no pain), 1–3 (mild pain), 4–6 (moderate pain), and 7–10 (severe pain). Abbreviations: *N* = total number of ALS participants; *n* = count; %, percentage; ALSAQ-5, the 5-item amyotrophic lateral sclerosis assessment questionnaire; PHQ-4, the 4-item patient health questionnaire; SD, standard deviation.

**Table 2:** Correlations between clinical factors, HRQoL, and PHQ-4 score in ALS patients.

Variables	ALSAQ-5		PHQ-4	
	r_s_	*p* Value	r_s_	*p* Value
Age	0.169	0.009	0.181	0.005
Time since first symptom	0.274	<0.001	0	0.998
Time required from symptom onset to diagnosis	0.101	0.122	0.089	0.174
ALS severity	0.728	<0.001	0.399	<0.001
Fatigue severity	0.473	<0.001	0.416	<0.001
Stress severity	0.480	<0.001	0.593	<0.001
Pain severity	0.425	<0.001	0.393	<0.001
Respiratory support	0.464	<0.001	0.197	0.002

Abbreviations: ALS, amyotrophic lateral sclerosis; ALSAQ-5, the 5-item amyotrophic lateral sclerosis assessment questionnaire; PHQ-4, the 4-item patient health questionnaire; HRQoL, health-related quality of life; r_s_, Spearman’s rank correlation coefficient.

**Table 3:** Predictors of HRQoL and psychological well-being in ALS patients.

Scales	Variables	Adjusted R^2^	β	95% CI	*p* Value	VIF
ALSAQ-5	Age	0.3832	0.17850	(0.0078, 0.3493)	0.0405	1.0509
	Time Since First Symptom Onset		0.22410	(0.1053, 0.343)	0.0003	1.0243
	Fatigue Severity		4.03000	(1.1411, 6.9205)	0.0065	1.6859
	Stress Severity		4.83960	(2.3901, 7.2893)	0.0001	1.6937
	Pain Severity		1.78340	(0.9061, 2.6609)	<0.0001	1.3658
PHQ-4	Age	0.3824	0.04640	(0.011, 0.0819)	0.0105	1.0078
	Stress Severity		2.00530	(1.5519, 2.4588)	<0.0001	1.2898
	Pain Severity		0.26270	(0.0824, 0.4431)	0.0045	1.2824

The final multiple linear regression analysis revealed that demographic and clinical variables influence HRQoL and psychological health in patients with ALS. Variables with significant correlations (*p* < 0.05) in the univariate analysis or those deemed clinically relevant were included in the multivariate model to perform multiple linear regression analysis. Abbreviations: ALS, amyotrophic lateral sclerosis; ALSAQ-5, the 5-item amyotrophic lateral sclerosis assessment questionnaire; β, unstandardized coefficient; CI, confidence interval; PHQ-4; 4-item patient health questionnaire; HRQoL, health-related quality of life; R, correlation coefficient; VIF, variance inflation factor.

## Data Availability

Informed consent was obtained from the participants and not authorized for public sharing of individual-level data. The data are available upon request by submitting a research proposal to the corresponding author, Dr. Guanqiao Li (guanqiaoli@tsinghua.edu.cn).

## Appendix A

**Table A1:** HRQoL and psychological well-being according to different demographics and clinical characteristics.

Variables	ALSAQ-5			PHQ-4		
	*n*	Median (Q1, Q3)	*p* Value	*n*	Median (Q1, Q3)	*p* Value
Age Group			0.068	15	5.00 (0.00, 9.00)	0.023 *
18–35	15	60.00 (44.00, 80.00)		37	4.00 (3.00, 8.00)	
36–45	37	60.00 (52.00, 76.00)		83	4.00 (2.00, 8.00)	
46–55	83	56.00 (48.00, 76.00)		67	6.00 (3.00, 10.00)	
56–65	67	64.00 (52.00, 84.00)		35	8.00 (4.00, 12.00)	
Greater than 65	35	76.00 (56.00, 88.00)		15	5.00 (0.00, 9.00)	
Sex			0.368			0.496
Male	139	60.00 (48.00, 80.00)		139	5.00 (2.00, 10.00)	
Female	98	64.00 (52.00, 84.00)		98	6.00 (3.00, 9.00)	
Type of ALS Diagnosis			0.248			0.067
Sporadic ALS	141	60.00 (52.00, 80.00)		141	4.00 (3.00, 8.00)	
Familial ALS	7	48.00 (32.00, 64.00)		7	3.00 (0.00, 6.00)	
Not sure	89	64.00 (48.00, 84.00)		89	6.00 (3.00, 12.00)	
Time Since First Symptom						
Less than 6 months	8	64.00 (40.00, 84.00)	<0.001 *	8	7.00 (2.50, 10.00)	0.879
6 to 12 months	23	60.00 (48.00, 80.00)		23	5.00 (4.00, 10.00)	
12 to 18 months	49	52.00 (44.00, 60.00)		49	4.00 (2.00, 8.00)	
18 months to 2 years	46	62.00 (48.00, 76.00)		46	4.00 (3.00, 9.00)	
2 to 3 years	50	72.00 (56.00, 88.00)		50	6.00 (3.00, 12.00)	
3 to 5 years	43	72.00 (56.00, 88.00)		43	6.00 (2.00, 9.00)	
More than 5 years	18	62.00 (52.00, 88.00)		18	5.00 (3.00, 7.00)	
Time Required from Symptom Onset to Diagnosis			0.47			0.246
Less than 3 months	11	56.00 (44.00, 84.00)		11	4.00 (0.00, 8.00)	
3 to 6 months	54	56.00 (48.00, 80.00)		54	4.00 (2.00, 10.00)	
6 to 12 months	90	64.00 (48.00, 76.00)		90	5.00 (3.00, 9.00)	
12 to 18 months	45	64.00 (48.00, 80.00)		45	5.00 (2.00, 8.00)	
18 months to 2 years	16	72.00 (52.00, 86.00)		16	7.00 (3.00, 10.50)	
2 to 3 years	9	80.00 (68.00, 88.00)		9	12.00 (7.00,12.00)	
ALS Severity			<0.001 *			<0.001 *
1—Able to work or perform housework	24	44.00 (32.00, 52.00)		24	3.50 (0.00, 7.50)	
2—Independent living but unable to work	79	48.00 (44.00, 56.00)		79	4.00 (1.00, 6.00)	
3—Requiring assistance for eating, excretion, or ambulation	85	72.00 (60.00, 84.00)		85	6.00 (4.00, 9.00)	
4—Presence of respiratory insufficiency, difficulty in coughing out sputum, or dysphagia	35	88.00 (72.00, 96.00)		35	10.00 (5.00, 12.00)	
5—Using a tracheostomy tube, tube feeding, or tracheostomy positive-pressure ventilation	14	88.00 (84.00, 92.00)		14	11.50 (8.00, 12.00)	
Fatigue Severity			<0.001 *			<0.001 *
Not fatigued	22	48.00 (40.00, 60.00)		22	2.00 (0.00, 5.00)	
Mildly fatigued	91	52.00 (44.00, 68.00)		91	4.00 (2.00, 7.00)	
Moderately fatigued	84	64.00 (56.00, 76.00)		84	6.00 (4.00, 9.00)	
Very fatigued	40	88.00 (80.00, 92.00)		40	12.00 (7.00, 12.00)	
Stress Severity			<0.001 *			<0.001 *
None	29	52.00 (40.00, 60.00)		29	0.00 (0.00, 3.00)	
Mild	90	56.00 (44.00, 68.00)		90	4.00 (2.00, 6.00)	
Moderate	81	68.00 (56.00, 84.00)		81	7.00 (4.00, 10.00)	
High	21	84.00 (72.00, 92.00)		21	10.00 (7.00, 12.00)	
Maximum	16	90.00 (82.00, 98.00)		16	12.00 (12.00, 12.00)	
Pain Severity			<0.001 *			<0.001 *
No pain	67	52.00 (44.00, 64.00)		67	3.00 (0.00, 7.00)	
Mild pain	95	60.00 (48.00, 76.00)		95	4.00 (3.00, 9.00)	
Moderate pain	55	76.00 (60.00, 88.00)		55	7.00 (4.00, 10.00)	
Severe pain	20	88.00 (76.00, 94.00)		20	10.50 (5.50, 12.00)	
ALS Treatment Drugs			0.103			0.376
Riluzole only	115	68.00 (48.00, 80.00)		115	5.00 (3.00, 10.00)	
Edaravone only	4	48.00 (44.00, 56.00)		4	4.50 (2.50, 5.50)	
Both riluzole and edaravone	88	58.00 (48.00, 78.00)		88	4.00 (2.00, 8.00)	
None	30	62.00 (56.00, 84.00)		30	5.00 (3.00, 12.00)	
Respiratory Support			<0.001 *			0.004 *
No respiratory support	140	56.00 (44.00, 70.00)		140	4.00 (2.00, 8.00)	
Intermittent use of non-invasive ventilator support	39	76.00 (56.00, 88.00)		39	4.00 (3.00, 11.00)	
Continuous use of non-invasive ventilator support during the night	30	70.00 (56.00, 84.00)		30	4.50 (2.00, 8.00)	
Continuous use of non-invasive ventilator support during day and night	16	86.00 (70.00, 94.00)		16	8.50 (5.00, 12.00)	
Use of invasive mechanical ventilation by intubation or tracheostomy	12	88.00 (84.00, 94.00)		12	11.50 (8.00, 12.00)	

Group differences were examined using the Kruskal–Wallis H test. Complete values were available for all variables (*N* = 237). ALS, amyotrophic lateral sclerosis; ALSAQ-5, 5-item amyotrophic lateral sclerosis assessment questionnaire; PHQ-4, 4-item patient health questionnaire; HRQoL, health-related quality of life; *N*, total number of included participants; *n*, count; Q1, first quartile; Q3, third quartile; * statistically significant.
